# High‐pressure homogenization and pH‐shifting modification of hazelnut protein isolates: Functional enhancement, allergenicity reduction, and probiotic microencapsulation performance

**DOI:** 10.1002/jsfa.70675

**Published:** 2026-04-19

**Authors:** Ilyas Atalar, Hatice Elen, Osman Gul, Abdullah Kurt, M.Irfan Aksu, Nevzat Konar

**Affiliations:** ^1^ Agriculture Faculty, Food Engineering Department Eskisehir Osmangazi University Eskisehir Türkiye; ^2^ Tayas Food R&D Center Gebze Türkiye; ^3^ Engineering and Architecture Faculty, Food Engineering Department Kastamonu University Konya Türkiye; ^4^ Aksehir Engineering and Architecture Faculty, Food Engineering Department Selcuk University Konya Türkiye; ^5^ Chemical and Metallurgical Engineering Faculty, Food Engineering Department Yildiz Technical University Istanbul Türkiye; ^6^ Agriculture Faculty, Food Engineering Department Ataturk University Erzurum Türkiye; ^7^ Agriculture Faculty, Dairy Technology Department Ankara University Ankara Türkiye

**Keywords:** high‐pressure homogenization treatment, hazelnut protein isolate, pH modification, allergenicity, probiotic encapsulation

## Abstract

**BACKGROUND:**

Plant‐based proteins, such as hazelnut protein isolates (HPIs), often exhibit limited solubility and functionality, as well as allergenic potential, thereby limiting their applicability in food products. This investigation sought to improve the techno‐functional characteristics of HPI and mitigate its allergenicity via high‐pressure homogenization treatment (HPHT) coupled with pH shifting, and to assess the feasibility of the modified HPI as a wall material for probiotic encapsulation.

**RESULTS:**

The combined application of alkaline pH (pH 12) and high pressure (875 bar) substantially enhanced protein solubility (up to 86.5%) and emulsion activity; concurrently, the zeta potential became more negative, suggesting increased electrostatic repulsion. Structural investigations demonstrated significant conformational alterations, including the disruption of α‐helix and β‐sheet structures and an increase in random coils, which, in turn, exposed hydrophobic groups and augmented surface hydrophobicity. These structural changes, crucially, modified protein epitopes, leading to a 49% decrease in HPI allergenicity. Furthermore, when assessed as a wall material for spray‐drying microencapsulation of *Lactobacillus acidophilus*, the modified HPI, in conjunction with maltodextrin (1:1 ratio), displayed the greatest protective capacity, preserving 7.96 log CFU mL^−1^ viability post‐drying and 7.22 log CFU mL^−1^ survival under simulated gastrointestinal conditions.

**CONCLUSION:**

The findings demonstrate that altering the structure of the HPI via HPHT and pH modulation enhances its solubility and emulsifying characteristics while simultaneously reducing its allergenic potential, thus supporting its use as a viable wall material. As a result, this approach offers a potentially beneficial method for converting hazelnut meal by‐products into adaptable, hypoallergenic ingredients for functional food applications. © 2026 The Author(s). *Journal of the Science of Food and Agriculture* published by John Wiley & Sons Ltd on behalf of Society of Chemical Industry.

## INTRODUCTION

Hazelnuts (*Corylus avellana*) are globally significant tree nuts, prized for their dense nutritional profile, which includes high‐quality oils, proteins, and essential vitamins. The industrial extraction of hazelnut oil, particularly through cold‐pressing, generates a protein‐dense, defatted meal as a by‐product, typically containing 30–50% protein.^1^, ^2^ This by‐product is often underutilized, frequently used as animal feed or discarded, resulting in a significant loss of valuable plant protein. Given the growing global demand for sustainable, plant‐based protein sources, converting hazelnut meal into high‐value protein isolates could be a valuable strategy to enhance the economic sustainability of the hazelnut oil sector.^3^ This approach would also help diversify the available plant protein options. However, the direct application of hazelnut protein isolates (HPIs) in modern food systems faces significant technical and safety challenges. The major subfractions of hazelnut protein can vary based on hazelnut cultivar and origin and hazelnut proteins contain higher ratios of 11S and 7S globulin subfractions than the other subfractions.^4^ These globulins are distinguished by their high molecular weight and intricate quaternary structures, maintained by disulfide bonds and hydrophobic interactions. Although hazelnut proteins possess a beneficial amino acid composition, abundant in arginine, glutamic acid, and branched‐chain amino acids,^2^ their functional characteristics, encompassing solubility, emulsifying ability, foaming capacity, and gelation, are intrinsically constrained in their native form. These limitations severely restrict their direct application in modern food systems, including plant‐based beverages, meat analogues, emulsions, and foamed products, where high solubility and interfacial activity are essential. Moreover, the presence of allergenic epitopes, off‐flavors, and anti‐nutritional compounds further limits their direct incorporation into food systems.^5^ To overcome these challenges, it is crucial to employ effective modification strategies that improve the techno‐functional properties of plant proteins, reduce or eliminate allergenicity and undesirable flavors, and tailor their characteristics to meet the specific requirements of diverse food applications.^6^ Generally, physical, chemical, and biological methods are employed to modify plant proteins. Physical modifications encompass thermal and non‐thermal processes. High‐pressure homogenization treatment (HPHT) is one of the most common non‐thermal modification techniques, inducing turbulence and cavitation that result in changes in particle size and protein conformation.^7^ The main advantages of HPHT systems include safety, low cost, high efficiency, and the absence of chemical materials. This technology involves passing a liquid through a narrow orifice at a controlled pressure, where excessive cavitation and shear forces are generated as the liquid passes through the homogenizer device. These effects can significantly alter functional properties, such as solubility and emulsion characteristics. Another widely employed strategy is pH shifting for modifying protein functionality. Altering the pH of proteins causes the molecular structure to unfold due to electrostatic repulsion.^8^, ^9^ Applying HPHT under different pH conditions can improve the techno‐functional and rheological properties of plant proteins.^9^, ^10^ In a recent study, Yildiz and Yıldız^11^ studied the effects of HPHT, pH treatment, and their combinations on quinoa proteins. HPHT following pH 12 adjustment effectively improved solubility and surface hydrophobicity. Comparable findings were reported for pine kernel proteins. The pH 12 adjustment was the most efficient treatment for increasing solubility, β‐sheet, random coils content, and the exposure of free sulfhydryl and hydrophobic groups.^12^ The combination of HPHT and pH shifting significantly affected the structure and techno‐functional properties of soy protein isolates. HPHT in alkaline or acidic solutions resulted in the formation of smaller reaggregated proteins and altered the rheological characteristics.^8^ Despite the potential of these two‐step methods, a notable gap remains in the current research. Although investigations have examined the effects of high‐pressure high‐temperature treatments on diverse legumes and seeds, the synergistic influence of HPHT and pH‐shifting on the structural and functional transformations of HPIs remains unexplored. Furthermore, there is a dearth of empirical evidence on the specific effects of these structural alterations on hazelnut allergenicity or on the efficacy of HPI as a biopolymeric wall material for probiotic delivery systems. The novelty of the study stems from its pioneering systematic assessment of the combined effects of HPHT and pH adjustment on HPI using response surface methodology (RSM). This study transcends fundamental functional analysis by (i) measuring the decrease in allergenic properties via structural alterations, and (ii) assessing the practical utility of modified HPI as a protective matrix for the spray‐drying microencapsulation of *Lactobacillus acidophilus*. Consequently, by addressing the dual issues of functionality and allergenicity, this research aims to develop a valuable, hypoallergenic application for hazelnut processing by‐products in functional foods.

## MATERIALS AND METHODS

### Materials

Blanched hazelnuts (*C. avellana*, Tombul variety) were supplied by Yavuzkan Hazel Gıda (Giresun), Türkiye, and oil was cold‐pressed at Ugurluoglu Company (Konya), Türkiye. Hazelnut meals contain 73.9 g kg^−1^ dry matter, 380.7 g kg^−1^ protein, 28.5 g kg^−1^ ash, 256.6 g kg^−1^ fat, 308.1 g kg^−1^ carbohydrate. HPI was prepared as described in the Extraction and HPHT–pH modification of hazelnut protein isolates section, with a composition of 974.0 ± 0.4 g kg^−1^ dry matter, 803.3 ± 1.3 g kg^−1^ protein, 112.1 ± 1.3 g kg^−1^ fat, 19.5 ± 0.2 g kg^−1^ ash, 48.5 ± 1.8 g kg^−1^ carbohydrate. Moisture, protein, fat, and ash analyses for hazelnut meal and HPI were performed using AOAC (2000) methods.

### Study design

The study comprised two stages: (i) modification of HPIs by HPHT combined with pH shifting, and (ii) evaluation of modified HPI as a wall material for probiotic microencapsulation via spray drying. In stage one, RSM with a central composite design (CCD) was used to examine the effects of pH (pH 6.0–12.0) and pressure (350–1400 bar) on HPI properties through 14 runs, including factorial, axial, and central points. An untreated sample served as a control (C1) and was neutralized at pH 7 and is coded as C2. Protein solubility, foaming and emulsifying capacities, particle size, zeta potential, rheology, and secondary structure were analyzed. In stage two, selected modified HPI were tested as wall materials for *L. acidophilus* encapsulation using spray drying. A full factorial design with inlet temperature (130 and 160 °C) and wall composition (HPI/maltodextrin (MD) ratios of 1:0, 1:1, 0:1) yielded six trials, alongside a non‐encapsulated control. Encapsulation efficiency, cell viability after drying, and survival under simulated gastrointestinal conditions were evaluated.

### Extraction and HPHT–pH modification of hazelnut protein isolates

Hazelnut meal pellets from cold pressing were ground into powder (Waring 8011S; Stamford, CT, USA) and mixed with distilled water (1:12, *w/v*). The mixture was homogenized (Ultra Turrax, IKA‐Werke GmbH & Co., T18; KG Staufen, Germany) at 7600 × *g* for 3 min, and the pH was adjusted to pH 12 with 5 mol L^−1^ sodium hydroxide (NaOH) under stirring. After centrifugation (Hermle Labortechnik GmbHZ 326K, Wehingen, Germany) at 7600 × *g* for 15 min at 4 °C, the residue was discarded, and the extraction was repeated three times. The combined supernatants were adjusted to pH 4.5 to precipitate proteins, then centrifuged again under the same conditions. The precipitate was freeze‐dried (Teknosem Toros TRS‐4/4V; Türkiye) to obtain protein isolate powders.^13^ This extraction process is selective for globular proteins, particularly enriching the 11S (legumin‐like) and 7S (vicilin‐like) globulin fractions, which are the major storage proteins in hazelnuts. The water‐soluble albumin fraction is largely removed during the washing and extraction steps. For protein modification, HPI was dispersed in purified water to obtain a 60 g L^−1^ protein solution. The pH was adjusted with NaOH or hydrochloric acid (HCl) as specified in the design (Table 1), and the suspension was homogenized at different pressures using a high‐pressure homogenizer (Panda PLUS 2000; GEA Niro Soavi, Parma, Italy). After homogenization, samples were frozen at −24 °C for 24 h and freeze‐dried.

**Table 1:** The techno‐functional properties of control and modified hazelnut protein isolates

Sample	pH	Pressure (bar)	Solubility (%)	Emulsion activity (m^2^ g^−1^)	Emulsion stability (min)	Foam capacity (%)	Foam stability (%)
C1	4.5	—	5.13 ± 0.13^j^	7.88 ± 0.67^h^	76.2 ± 0.33^a^	1.49 ± 1.45^i^	ND
C2	7	—	25.13 ± 0.16^i^	20.93 ± 0.54^g^	37.7 ± 0.21^e^	5.40 ± 0.51^h^	ND
1	9	875	66.96 ± 1.45^fg^	26.99 ± 1.23^bc^	31.22 ± 0.18^g^	16.22 ± 0.02^ef^	71.42 ± 2.38^cd^
2	11	1250	74.53 ± 1.39^cde^	24.91 ± 1.12^de^	45.21 ± 0.19^bc^	34.29 ± 0.11^b^	83.33 ± 1.29^b^
3	11	500	61.95 ± 1.33^gh^	30.07 ± 1.46^ab^	48.17 ± 0.23^b^	21.13 ± 0.03^cd^	73.33 ± 1.15^c^
4	9	1400	82.50 ± 5.94^ab^	24.56 ± 1.33^e^	27.61 ± 0.15^h^	11.11 ± 0.07^g^	56.90 ± 1.34^f^
5	9	875	76.76 ± 3.27^bcd^	23.05 ± 2.01^ef^	41.84 ± 0.27^d^	12.92 ± 0.12^fg^	68.89 ± 0.98^de^
6	9	875	78.27 ± 4.75^bc^	24.02 ± 1.12^e^	46.94 ± 0.33^bc^	20.06 ± 0.16^cd^	67.76 ± 2.21^e^
7	6	875	10.64 ± 1.79j	28.17 ± 1.46^abc^	25.73 ± 0.17^h^	14.71 ± 0.26^f^	65.73 ± 0.45^e^
8	9	875	72.57 ± 4.64^def^	24.04 ± 1.23^e^	38.20 ± 0.04^e^	21.21 ± 0.33^c^	71.65 ± 0.03^cd^
9	9	350	86.50 ± 3.81^a^	27.10 ± 1.26^bc^	35.21 ± 0.18^f^	16.22 ± 0.28^ef^	66.66 ± 1.23^e^
10	7	500	34.12 ± 2.38^h^	22.38 ± 0.67^f^	35.97 ± 0.16^f^	13.04 ± 0.62^fg^	55.55 ± 2.76^f^
11	12	875	84.44 ± 2.89^a^	33.12 ± 0.33^a^	31.78 ± 0.33^g^	49.44 ± 0.14^a^	92.85 ± 1.45^a^
12	9	875	64.83 ± 2.68^g^	25.00 ± 0.56^de^	40.00 ± 0.01^de^	20.68 ± 0.26^cd^	65.89 ± 2.23^e^
13	9	875	68.86 ± 1.74^efg^	24.00 ± 0.01^e^	41.00 ± 0.02^d^	21.12 ± 0.23^c^	71.65 ± 1.12^cd^
14	7	1250	25.35 ± 1.17^i^	25.07 ± 0.23^de^	30.53 ± 0.06^g^	22.22 ± 0.36^c^	81.25 ± 2.23^b^

*Note*: Values are given as mean ± standard deviation from triplicate determinations. Means within the same column with different superscript letters are significantly different (*P* < 0.05). C1, hazelnut protein isolates at pH 4.5; C2, hazelnut protein isolates at pH 7.0 (neutral pH); ND, not determined.

### Determination of techno‐functional properties

#### Solubility

The solubility of modified and control HPI was determined following Klompong *et al*.^14^ Protein suspensions were centrifuged at 6000 × *g* for 15 min at 4 °C. The supernatant was mixed 1:1 (*v/v*) with Biuret reagent and measured at 500 nm using an ultraviolet (UV)‐spectrophotometer (Jasco V‐730; Jasco, Tokyo, Japan). Protein content was calculated from a bovine serum albumin standard curve (*Y =* 0.1017*X +* 0.0749, coefficient of determination (*R* ^2^) = 0.991), and solubility was expressed using Eqn (1).

(1) $$\text{Solubility}\;\left(\%\right)\;=\frac{\text{Supernatant protein amounts}}{\text{Total protein amounts}}\times 100$$

#### Emulsion and foaming properties

The emulsion activity and stability of control and modified HPIs were determined according to the methodology developed by Pearce and Kinsella.^15^ Absorbance was recorded at 500 nm using a UV‐spectrophotometer (Jasco V‐730; Jasco), and values were calculated with Eqns (2) and (3).

(2) $$\text{Emulsion activity index}\;\left(\mathrm{EAI}\right)=\frac{2\times 2.303\times A_{0}}{0,25\times \text{sample amount}}$$

(3) $$\text{Emulsion stability index}\;\left(\mathrm{ESI}\right)=\frac{A_{10}\times \Delta t}{\Delta A}$$

where *A*_0_ is the absorbance value immediately after emulsion formation, *A*_10_ is the absorbance value 10 min after emulsion formation, Δ*t* = 10 min, and Δ*A* is the difference between the absorbance value at initial formation and 10 min later.

The foam‐forming properties of hazelnut proteins were determined based on the method proposed by Ogunwolu *et al*.^16^ Samples were homogenized at 14 000 rpm for 3 min, and foam volume was recorded immediately and after 30 min to calculate foam capacity and stability using Eqns (4) and (5).

(4) $$\text{Foam capacity}\;\left(\%\right)=\frac{V2-V1}{V1}\times 100$$

(5) $$\text{Foam stability}\;\left(\%\right)=\left(V30-V1\right)/\left(V2-V1\right)\times 100$$

where, V2 represents the volume after homogenization (in milliliters), V1 the volume before homogenization (in milliliters), and V30 the volume after 30 min of homogenization (in milliliters).

### Determination of rheological parameters

#### Steady shear test

The flow behavior of the control sample and modified HPIs at 25 ± 0.1 °C was determined using a rheometer (Haake Mars 40, Thermo Scientific, Karlsruhe, Germany) with a 2° cone‐plate geometry (35 mm diameter). Suspensions containing 40 g L^−1^ protein were tested for 300 s at shear rates of 0–100 s^−1^.

#### Dynamic shear test

Dynamic rheological tests were conducted to evaluate the viscoelastic properties of HPI. Suspensions (4% protein) were prepared in water, and stress sweep analysis (0.01–10.0 Pa, 1.00 Hz) was used to determine the linear viscoelastic region (0.20 Pa). Frequency sweep was then carried out from 0.00 to 10.0 Hz at 25.0 ± 0.1 °C. The complex modulus (G*) was calculated using Eqn (6):

(6) $$G^{*}=\sqrt{{\left(G\prime \right)}^{2}+{\left(G^{\prime \prime }\right)}^{2}}$$

where, G* is the complex modulus (in pascals), *G'* is the storage modulus (in pascals), and *G”* is the loss modulus (in pascals).

### Determination of structural properties

#### Particle size

Particle size distribution of HPI dispersions (4.00%) was measured by laser diffraction (Mastersizer 2000; Malvern Instruments, Malvern, UK). Samples were diluted 1:100 with ultrapure water and analyzed at 25 °C.

#### Zeta potential

Zeta potential of control and modified HPI was measured using a Zetasizer Nano ZS90 (Malvern Instruments). Samples were prepared in distilled water at a concentration of 1 mg mL^−1^, and 1 mL of the solution was injected into the zeta cell.

#### Secondary structures

Chemical bonding changes in powdered HPI were analyzed by Fourier‐transform infrared (FTIR) spectroscopy (Bruker Alpha II; Bruker, Karlsruhe, Germany) over the range of 4000–650 cm^−1^ at a resolution of 4.00 cm^−1^. Secondary structures (α‐helix, β‐sheet, β‐turn, random coil) were identified from the amide I region (1600–1700 cm^−1^). Peakfit 4.12 software was used to calculate the secondary structure content of HPI. The correspondence between each sub‐peak and secondary structure is as follows: α‐helix is 1648–1664 cm^−1^, β‐sheet is 1615–1637 cm^−1^ and 1682–1700 cm^−1^, β‐turn is 1664–1681 cm^−1^, and random coils is 1637–1648 cm^−1^.^7^

#### Surface hydrophobicity

The surface hydrophobicity of protein suspensions was determined using the method proposed by Arzeni *et al*.^17^ with a fluorescent spectrophotometer (Horiba Scientific, Kyoto, Japan). The fluorescence intensity of protein suspensions was recorded at 390/468 nm at 25 °C, and the hydrophobicity index was calculated from the initial slope of the relative fluorescence intensity (RFI) *versus* protein concentration, using Eqn (7).^18^ Prior to the 8‐anilinonaphthalene‐1‐sulfonic acid (ANS) binding assay, all protein samples were diluted to a standard, neutral pH (pH 7.0) to ensure that the fluorescence measurements were conducted under identical pH conditions for both the control and the treated samples. While this neutralization cancels the immediate effect of the medium pH on ANS fluorescence intensity, it enables quantification of permanent structural rearrangements. The resulting hydrophobicity index, therefore, reflects the irreversible unfolding and exposure of hydrophobic residues that occurred during the alkaline‐HPHT treatment and remained accessible upon returning to neutral conditions.

(7) $$\;\mathrm{RFI}=\frac{F-F_{0}}{F_{0}}$$

where, *F* represents the value of protein–ANS fluorescent reading, *F*_0_ the value of ANS solution reading without protein.

### Characterization of optimum HPI sample

#### Allergenicity

Allergenicity of HPI was assessed using a Veratox Hazelnut Allergen Antibody enzyme‐linked immunosorbent assay (ELISA) kit. Analyses were performed according to the manufacturer's instructions, and absorbance was measured at 450 nm using an ELISA Reader (SH‐1000; Corona Electric, Hitachinaka, Japan). Results were calculated from the standard curve (*Y =* 0.1067*X +* 0.8608, *R* ^2^ = 0.9379).

#### Surface microstructure

Surface microstructure of modified HPI was examined by scanning electron microscopy (SEM) (JSM‐7001F; Jeol, Akishima, Tokyo, Japan). Samples were coated with a 10 nm gold–palladium layer (Quorum SC7620; Laughton, UK), and images were captured at magnifications of 500× and 2000×.

### Usage of optimum HPI for probiotic encapsulation

#### Bacterial enrichment and spray‐drying encapsulation

For bacterial enrichment, 0.5 g of *L. acidophilus* powder was inoculated into 5 mL de Man, Rogosa and Sharpe (MRS) broth and incubated at 37 °C for 24 h, then transferred to 95 mL fresh broth and incubated for another 24 h. Biomass was collected by centrifugation (4000 × *g*, 10 min, 4 °C), washed twice with sterile isotonic sodium chloride (NaCl), and resuspended in the same solution. Bacterial counts were determined on MRS agar after 120 h at 37 °C and expressed as log colony‐forming unit (CFU) per milliliter. Enriched bacteria were mixed with a matrix containing different ratios of MD and HPI and encapsulated using a spray‐dryer (Unopex, İzmir, Türkiye). Three wall material ratios (1:0, 1:1, 0:1 *w/w*) and two inlet air temperatures (130 and 160 °C) were studied in the encapsulation process based on preliminary studies. Mixtures were spray‐dried in a laboratory‐scale dryer at inlet temperatures of 130 and 160 °C. Before drying, 9.76–9.85 log CFU mL^−1^ of the enriched culture was added to the solution.

#### Determination of probiotic viability under simulated gastrointestinal conditions

Survival of encapsulated *L. acidophilus* was tested under simulated gastrointestinal conditions using the standardized static *in vitro* digestion protocol of Brodkorb *et al*.,^19^ which includes oral, gastric, and intestinal phases.

- Oral phase: 1 g of encapsulated bacteria was mixed with simulated salivary fluid (3.5 mL), α‐amylase (0.5 mL), calcium chloride (CaCl_2_, 25 μL), and distilled water (975 μL). After adjusting pH to pH 7.0 (NaOH), samples were incubated at 37 °C for 2 min.
- Gastric phase: simulated gastric fluid (6.0 mL), pepsin (1.28 mL), distilled water (556 μL), and CaCl_2_ (4 μL) were added, pH was adjusted to pH 3.0 (HCl) and incubated at 37 °C for 2 h. Then, 1 mL was sampled for viability counts.
- Intestinal phase: gastric chyme was combined with intestinal fluid (7.7 mL), pancreatin (3.5 mL), bile salts (1.75 mL), distilled water (917 μL), and CaCl_2_ (28 μL). After adjusting pH to pH 7.0 (NaOH), samples were incubated at 37 °C for 2 h, and 1 mL was taken for bacterial enumeration.

### Statistical analysis

A CCD under RSM was used to plan experimental runs. Effects of independent variables were assessed by analysis of variance (ANOVA) at *P* ≤ 0.05, with *P* > 0.05 considered non‐significant (Table 2). Tukey's *post hoc* test was applied to the results of all runs. RSM with a CCD was used to examine the effects of pH (pH 6.0–12.0) and pressure (350–1400 bar). The design, comprising 14 experimental runs, is presented in Table 1 (samples 1–14). Separately, an untreated HPI sample dispersed at pH 7.0 served as the sole control for baseline comparison. Multiple linear regression was applied to develop predictive models, and model adequacy was verified using *R* ^2^, lack‐of‐fit, and residual analyses. The models were then used to identify optimal conditions for HPI functionality and structure, allowing for the simultaneous evaluation of multiple responses. All analyses were performed with three independent replicates (*n* = 3), and results are presented as mean ± standard deviation. For the CCD, each experimental run was prepared and analyzed independently in triplicate. The adequacy of the fitted models was evaluated using *R* ^2^, adjusted‐*R* ^2^, and lack‐of‐fit tests. For some responses (e.g., solubility, apparent viscosity), the lack‐of‐fit test was significant (*P* < 0.05). This can sometimes occur with highly precise replicate measurements at center points, leading to a very small pure error estimate. While the significant lack‐of‐fit suggests the quadratic model may not capture all systematic variation, the high *R* ^2^ and adjusted‐*R* ^2^ values (> 0.70) indicate it still provides a useful and significant approximation of the response surface for the purpose of identifying major trends and optimal regions.

**Table 2:** The effects of process parameters on the techno‐functional properties of hazelnut protein isolates

Factors	Solubility (%)		Emulsion activity (m^2^ g^−1^)		Emulsion stability (min)		Foam capacity (%)		Foam stability (%)	
	Coefficient	*P*	Coefficient	*P*	Coefficient	*P*	Coefficient	*P*	Coefficient	*P*
	Quadratic		Quadratic		Linear		Quadratic		Quadratic	
Model	6251.74	0.0015	89.38	0.0126	202.8	0.1339	1028.08	0.0131	871.62	0.0554
A – pH	4111.89	0.0002	26.37	0.0168	156.93	0.0787	599.89	0.003	423.60	0.0190
B – Pressure (bar)	0.4216	0.9508	4.61	0.2439	45.87	0.3172	28.55	0.386	59.94	0.3023
AB	113.99	0.3256	15.41	0.0504			3.96	0.7415	61.62	0.2960
A^2^	1931.97	0.0026	42.73	0.005			325.26	0.0148	230.00	0.0628
B^2^	39.16	0.5564	8.47E‐06	0.9987			48.71	0.2653	74.40	0.2542
Lack‐of‐fit	684.89	0.0248	14.04	0.1711	326.82	0.2238	214.16	0.0387	365.08	0.0030
Pure error	146.58		9.25		132.75		57.49		29.35	
*R* ^2^	0.8826		0.7933		0.3062		0.791		0.6885	
Adjusted‐*R* ^2^	0.8092		0.6641		0.18		0.6604		0.4937	

## RESULTS AND DISCUSSION

### Techno‐functional properties

#### Solubility

**Figure 1:**
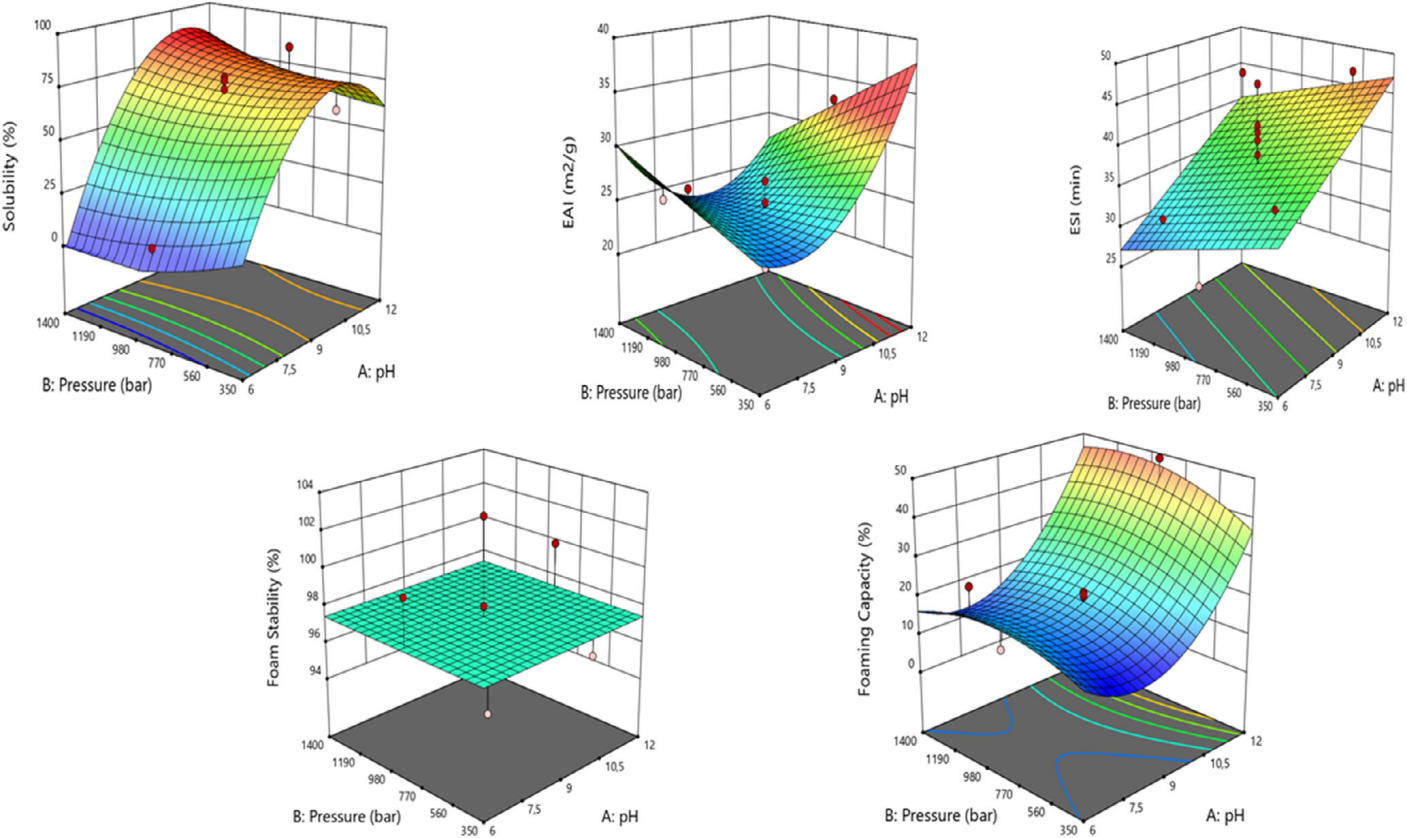
Three‐dimensional (3D) response surface plots illustrating the effects of pH and homogenization pressure on the techno‐functional properties of hazelnut protein isolates. EAI, emulsion activity index (m^2^ g^−1^); ESI, emulsion stability index (min).

Protein and protein isolate solubility is critical for their functionality in beverages, foams, and emulsion systems. The solubility values of HPI are presented in Table 1. The effect of pH on solubility was statistically significant (*P* < 0.05) (Table 2). Solubility was relatively low at low pH levels, while an increase in pH resulted in a corresponding increase in solubility. Notably, solubility approached maximum values in the pH 9–10 range (Fig. 1). An increase in pressure resulted in only a slight, non‐significant improvement in solubility (*P* > 0.05). The interactions among pressure intensity, protein source, and medium pH strongly influence the effect of high‐pressure treatment on protein solubility. Several studies demonstrate that neutral or alkaline pH combined with elevated pressure generally enhances solubility, whereas the opposite trend may occur under acidic conditions. For example, Navare *et al*.^20^ reported that lentil protein concentrate exhibited decreased solubility under pressure at pH 3, indicating that pressure‐induced conformational changes can intensify aggregation near the isoelectric point. In contrast, Wang *et al*.^21^ observed that hemp seed protein isolate subjected to increasing pressure at neutral pH showed marked improvements in solubility, particularly when pressure and pH were applied synergistically. Similarly, Karabulut *et al*.^22^ demonstrated that treating hemp protein isolate under alkaline conditions (pH 12) and 130 MPa (1300 bar) substantially enhanced its solubility compared to the untreated control. Taken together, these findings highlight that solubility outcomes are governed by the density of charged groups on the protein surface. At higher pH values, the ionization of amino acid side chains increases electrostatic repulsion, and pressure facilitates unfolding, thereby exposing hydrophilic residues to the aqueous environment. This mechanistic interplay suggests that strategic pH adjustment, in combination with pressure treatment, can be an effective approach to tailor solubility in diverse plant proteins. The increase in solubility is driven by two main factors: intense electrostatic repulsion from the high net negative charge at alkaline pH, and pressure‐induced unfolding that increases the protein surface area and exposes additional hydrophilic and charged residues that were previously less accessible or engaged in stabilizing internal bonds. This enhances hydration and protein–water interactions.

#### Emulsion and foam properties

Protein emulsifying properties mainly rely on reducing surface tension, preventing droplet coalescence, and improving interfacial behavior. These functions are primarily determined by surface characteristics rather than the overall hydrophobicity of the protein.^23^ Across all treatment groups, EAI values were significantly higher than those in the control, indicating that high‐pressure treatment, combined with pH adjustment, improved emulsifying capacity. The improvement in emulsification results from increased surface hydrophobicity arising from the exposure of non‐polar residues during the same unfolding process, thereby increasing adsorption at the oil–water interface. In contrast, the control sample exhibited poor emulsifying activity (EAI = 7.88 m^2^ g^−1^), indicating a low capacity to form and stabilize new oil–water interfaces. However, precisely because so few oil droplets were successfully formed, there were fewer droplets available to collide and coalesce. This resulted in a mechanically stable, albeit very coarse and inefficient, emulsion, which is reflected in the relatively high ESI (ESI = 76.20 min). Statistical analysis further revealed that the effect of pH on EAI was significant (*P* < 0.05) (Table 2). Under low pressure and high pH conditions, the EAI value increased. The increase in pressure across different pH conditions showed varying effects (Fig. 1). For instance, increasing pressure at lower pH led to higher EAI values, whereas at higher pH, it led to lower EAI values. In contrast, ESI generally exhibited a declining trend with increasing pressure, suggesting that while pressure can enhance protein unfolding and interfacial activity, it may simultaneously destabilize the formed emulsions. These findings showed a similar trend across all pH levels (Fig. 1). Increasing pH levels had a positive effect on ESI. Previous studies suggest that protein emulsifying properties are pressure‐dependent, with improvements observed up to a certain threshold followed by reductions at higher pressures. For example, Saricaoglu *et al*.^13^ reported that HPI exhibited significant increases in both EAI and ESI up to 100 MPa, whereas further pressurization to 150 MPa resulted in a decline. This reduction was attributed to excessive protein denaturation and subsequent aggregation, which hinders interfacial functionality. Similarly, Karabulut *et al*.^22^ demonstrated that modification of hemp protein isolate at pH 12 and 130 MPa enhanced EAI from 12 to 20 m^2^ g^−1^ and ESI from 15 to 32 min. The authors attributed these improvements to the formation of a strong viscoelastic protein film at the oil–water interface, which enhances both adsorption and resistance to coalescence. Collectively, these findings highlight that while moderate pressure facilitates unfolding and interfacial film formation, excessive pressure may induce aggregation and compromise emulsion stability.

The results for the foam‐forming properties of both control samples and modified HPI are presented in Table 1. The highest foam capacity was 49.44% at pH 12 and 875 bar. The increase in pH has led to higher foam capacity across all pressure conditions. No significant change in foam stability was observed for HPI subjected to high‐pressure treatment. Ma *et al*.^24^ applied high‐pressure treatment to chickpea protein isolate at different pressure levels (30, 60, 90, 120, and 150 MPa). After 30 MPa, the increase in high‐pressure treatment and the number of cycles led to a decrease in foam capacity values. Excessive pressure and cycles caused protein denaturation, leading to more irregular, larger foam bubbles and reduced foam stability.

### Flow behavior and viscoelastic characteristics

**Figure 2:**
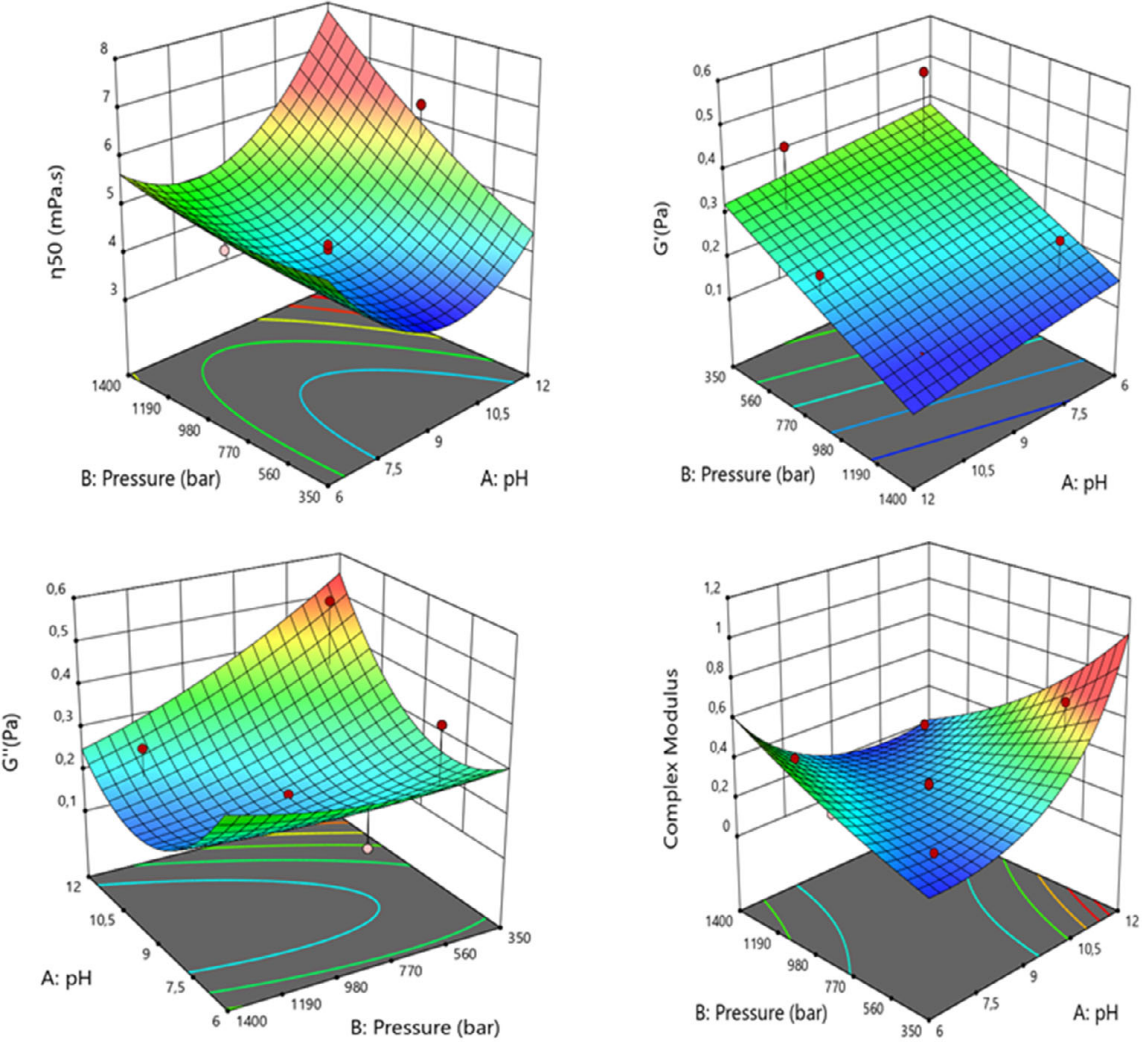
Three‐dimensional (3D) response surface plots illustrating the effects of pH and homogenization pressure on the rheological properties of hazelnut protein isolates. *η*_50_, apparent viscosity at 50 s^−1^; *G*′, storage modulus; *G*″, loss modulus; G*, complex modulus.

Table 3 summarizes the rheological properties of HPIs, including apparent viscosity (*η*_50_), *G*′, *G*″, and G*, measured at 1 Hz under different pH and pressure conditions. Both pH and pressure significantly affected apparent viscosity (*P* < 0.05) (Table 4). In general, apparent viscosity increased with increasing pressure^25^ (Fig. 2), and alkaline pH further enhanced this increase. This observation may seem counterintuitive, as a reduction in particle size often decreases viscosity. However, in protein dispersions, viscosity is governed not only by particle size but also critically by protein solubility, hydration, and intermolecular interactions.^26^, ^27^ The combined high‐pressure and high‐pH treatment markedly enhanced protein solubility, leading to a higher concentration of hydrated molecules in the continuous phase and thereby increasing the effective volume fraction. Furthermore, protein unfolding exposed hydrophobic and charged groups, promoting stronger protein–protein interactions that formed a weak, transient network, thereby increasing flow resistance and apparent viscosity despite the smaller particle size. Previous studies also indicate that the magnitude and direction of viscosity changes vary depending on protein source and treatment level. Huang *et al*.^28^ reported that chickpea protein viscosity decreased up to 30 MPa due to reduced frictional resistance from smaller particle size and network disruption but subsequently increased above 30 MPa before declining again at 120 MPa due to excessive pressure.

**Table 3:** Rheological properties of control and modified hazelnut protein isolates

Sample	pH	Pressure (bar)	*η*_50_ (mPa s)	*G*′ (Pa)	*G*″ (Pa)	G* (Pa)
C1	4.5	—	1.11 ± 0.01^j^	1.226 ± 0.022^a^	1.022 ± 0.013^a^	1,59 ± 0,02^a^
C2	7	—	1.14 ± 0.02^j^	0.180 ± 0.020^f^	0.292 ± 0.016^c^	0.34 ± 0.06^d^
1	9	875	4.08 ± 0.06^fg^	0.202 ± 0.018^e^	0.186 ± 0.012^ef^	0.27 ± 0.06^ef^
2	11	1250	5.34 ± 0.04^bc^	0.145 ± 0.009^g^	0.272 ± 0.014^cd^	0.31 ± 0.07^de^
3	11	500	3.59 ± 0.02^h^	0.453 ± 0.014^bc^	0.531 ± 0.006^b^	0.69 ± 0.04^b^
4	9	1400	5.73 ± 0.09^b^	0.136 ± 0.021^h^	0.113 ± 9.017^h^	0.178 ± 0.01^h^
5	9	875	4.06 ± 0.07^fg^	0.194 ± 0.006^ef^	0.187 ± 0.008^ef^	0.27 ± 0.01^ef^
6	9	875	3.98 ± 0.05^g^	0.198 ± 0.009^ef^	0.187 ± 0.005^ef^	0.27 ± 0.02^ef^
7	6	875	4.91 ± 0.03^cd^	0.263 ± 0.006^d^	0.199 ± 0.004^e^	0.33 ± 0.03^d^
8	9	875	4.05 ± 0.12^fg^	0.202 ± 0.008^e^	0.186 ± 0.006^ef^	0.19 ± 0.01^gh^
9	9	350	3.41 ± 0.07^i^	0.189 ± 0.015^f^	0.121 ± 0.018^g^	0.22 ± 0.03^fg^
10	7	500	4.55 ± 0.04^e^	0.514 ± 0.018^b^	0.385 ± 0.012^bc^	0.24 ± 0.02^f^
11	12	875	6.44 ± 0.01^a^	0.269 ± 0.015^cd^	0.240 ± 0.021^d^	0.36 ± 0.06^cd^
12	9	875	4.01 ± 0.01^g^	0.190 ± 0.013^f^	0.195 ± 0.009^e^	0.27 ± 0.03^ef^
13	9	875	4.18 ± 0.02^f^	0.190 ± 0.018^f^	0.186 ± 0.011^ef^	0.27 ± 0.02^ef^
14	7	1250	4.84 ± 0.05^d^	0.260 ± 0.014^d^	0.295 ± 0.016^c^	0.39 ± 0.01^c^

*Note*: Values are given as mean ± standard deviation from triplicate determinations. Means within the same column with different superscript letters are significantly different (*P* < 0.05). C1, hazelnut protein isolates at pH 4.5; C2, hazelnut protein isolates at pH 7.0 (neutral pH); *η*_50_, apparent viscosity at 50 s^−1^; *G*′, storage modulus; *G*″, loss modulus; G*, complex modulus.

**Table 4:** The effects of process parameters on the rheological properties of hazelnut protein isolates

Factors	*η*_50_ (mPa s)		*G*′ (Pa)		*G*″(Pa)		G* (Pa)	
	Coefficient	*P*	Coefficient	*P*	Coefficient	*P*	Coefficient	*P*
	Quadratic		Linear		Quadratic		Quadratic	
Model	7.80	0.0068	0.0540	0.0972	0.0607	0.4740	0.0513	0.0387
A – pH	0.3638	0.2219	0.0035	0.5530	0.0040	0.5799	0.0061	0.1585
B – Pressure (bar)	3.55	0.0033	0.0505	0.0399	0.0161	0.2823	0.0029	0.3141
AB	0.5302	0.1484			0.0072	0.4642	0.0227	0.0170
A^2^	3.32	0.0039			0.0326	0.1395	0.0193	0.0242
B^2^	0.1072	0.4926			0.0017	0.7149	0.0000	0.8954
Lack‐of‐fit	1.63	<0.0001	0.1021	<0.0001	0.0969	<0.0001	0.0165	0.0263
Pure error	0.0247		0.1021		0.0969		0.0036	
*R* ^2^	0.8246		0.3454		0.3850		0.7184	
Adjusted‐*R* ^2^	0.7150		0.2264		0.0007		0.5425	

*Note*: *η*_50_, apparent viscosity at 50 s^−1^; *G*′, storage modulus; *G*″, loss modulus; G*, complex modulus.

Dynamic shear tests were conducted to investigate the impact of HPHT and pH on the viscoelastic properties of HPI dispersions (Table 3). Elastic properties decreased with increasing pressure (*P* < 0.05) and were markedly reduced at alkaline pH compared to the acidic control, indicating weakening of the protein network. Viscosity properties showed less consistent changes, generally remaining lower than the control but exhibiting relative increases under specific pH–pressure combinations, particularly at pH 11 and moderate pressure. Consequently, viscous behavior (*G*″ ≥ *G′*) was dominant in most treated samples, especially under high‐pressure (> 875 bar) and alkaline pH conditions. Similar findings were reported by Baskıncı and Gul,^29^ who observed that sesame protein isolates subjected to high pressure displayed a higher viscous modulus than elastic modulus at low oscillation frequencies (10 rad s^−1^), with elastic behavior becoming dominant only at higher frequencies. At 50 MPa, the viscous modulus remained dominant across all frequencies, and both moduli decreased as pressure increased, depending on oscillation frequency.

The G* is a key parameter that describes the overall resistance of a material to deformation when considered as an elastic solid. In the present study, the untreated control exhibited the highest G* value (1.59 Pa), whereas all treated samples showed lower values, reflecting a reduction in structural rigidity after pressure modification (Table 3). The maximum G* (0.69 Pa) was observed among the treated groups at pH 11 and 500 bar, indicating that moderate pressure under alkaline conditions partially enhanced viscoelastic behavior. Statistical analysis confirmed that the interaction between pH and pressure significantly affected G* (*P* < 0.05) (Table 4). Interestingly, G* tended to increase with pressure under acidic conditions (pH 7), while the opposite trend was observed under alkaline conditions (pH 11–12), suggesting that pressure‐induced structural rearrangements are strongly modulated by the ionization state of amino acid residues (Fig. 2).

For instance, increasing the intensity of high‐pressure homogenization to 70 MPa for three passes has been shown to increase both the *G*′ and the complex viscosity, reflecting a more elastic, solid‐like behavior due to enhanced protein–protein network formation.^30^ Conversely, some studies indicate that high‐pressure processing can lead to a decrease in *G*′ and *G*″, particularly at higher pressures, suggesting a more fluid‐like behavior due to protein denaturation and disruption of aggregate structures.^31^ This variability underscores the critical dependence of viscoelastic properties on the specific high‐pressure parameters, protein concentration, and intrinsic protein characteristics.^32^

### Structural properties

#### Particle size

**Figure 3:**
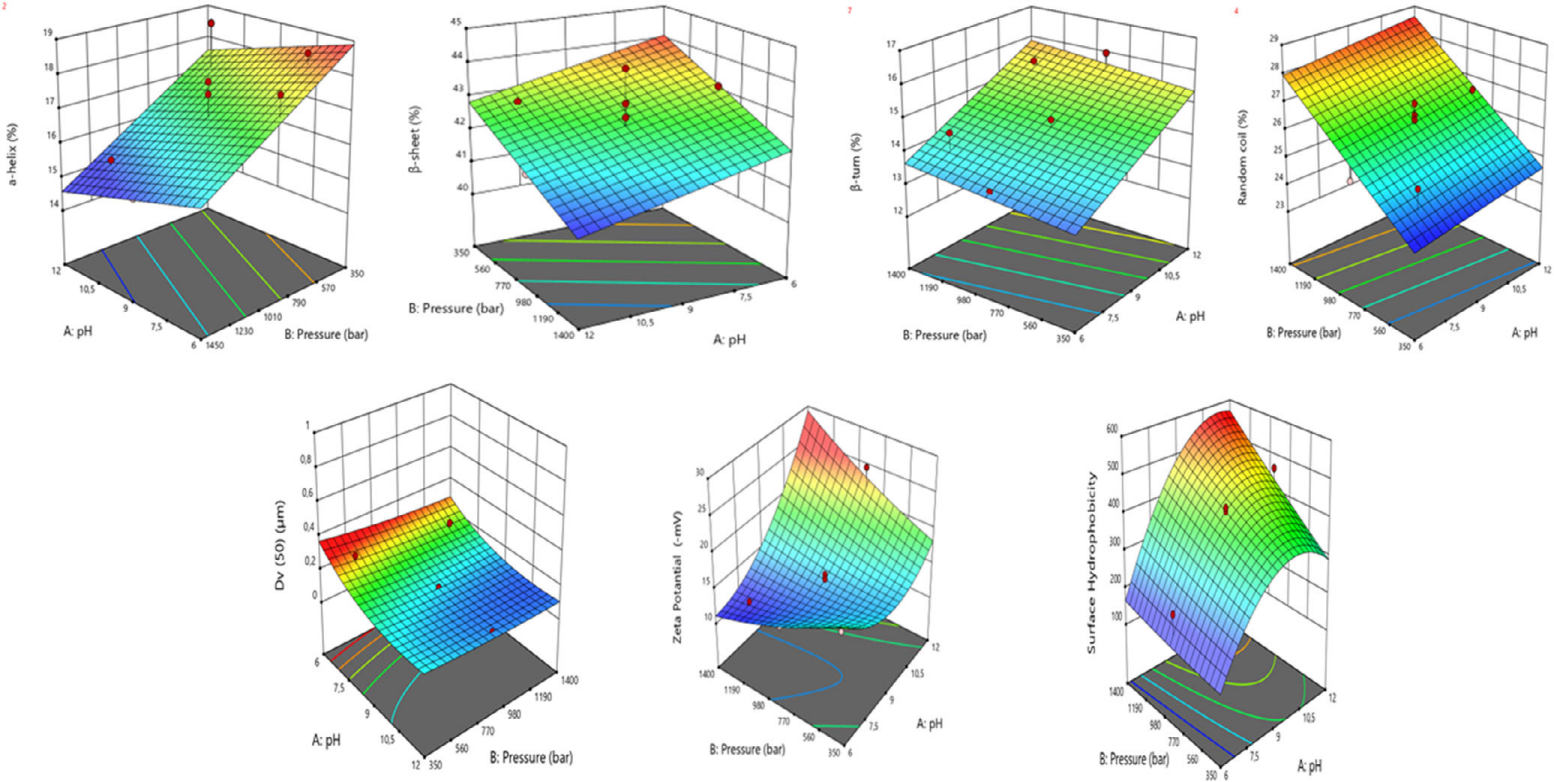
Three‐dimensional (3D) response surface plots illustrating the effects of pH and homogenization pressure on the structural properties of hazelnut protein isolates.

The particle size of protein aggregates directly affects their techno‐functional properties, such as emulsification, foaming, and gelation. The particle size (D50) results of HPIs are presented in Table 5. The effect of pH and pressure on D50 was significant (*P* < 0.05). Increasing pressure generally reduced D50, particularly at lower pH, while its effect was less pronounced at higher pH (Fig. 3). These reductions in particle size may be related to turbulent flow, shear stress, cavitation, pressure intensity, and pH, which together disrupt protein aggregates. Large protein aggregates can be broken down by HPHT, weakening non‐covalent interactions such as hydrophobic, hydrogen bonding, and electrostatic forces.^33^ Similar findings have been reported by Wang *et al*.,^21^ who showed that combining HPHT with pH shifting further reduced the particle size of chickpea proteins, as unfolded proteins generated by extreme pH were more susceptible to fragmentation and dissociation under intense external forces.

**Table 5:** Structural and conformational properties of control and modified hazelnut protein isolates

Sample	pH	Pressure (bar)	Average particle size (D50) (μm)	Zeta potential (mV)	α‐Helix (%)	β‐Sheet (%)	β‐Turn (%)	Random coil (%)	Surface hydrophobicity
C1	4.5	—	391 ± 27.33^a^	0.14 ± 0.01^a^	20.80 ± 0.12^ab^	44.81 ± 0.08^e^	11.84 ± 0.09^i^	22.54 ± 0.13^b^	120 ± 1.56^i^
C2	7	—	0.271 ± 0.03^bc^	−8.48 ± 0.06^b^	19.96 ± 0.35^b^	44.15 ± 0.22^e^	12.12 ± 0.15^hi^	23.13 ± 0.22^b^	168 ± 1.48^h^
1	9	875	0.140 ± 0.04^def^	−14.03 ± 0.07^de^	17.22 ± 0.03^cd^	45.23 ± 0.07^e^	13.19 ± 0.09^gh^	24.36 ± 0.07^ab^	420 ± 1.02^cd^
2	11	1250	0.085 ± 0.03^f^	−20.5 ± 0.21^g^	20.33 ± 0.06^b^	47.73 ± 0.14^d^	21.43 ± 0.12^bc^	10.51 ± 0.21^e^	480 ± 0.68^b^
3	11	500	0.121 ± 0.03^ef^	−14.03 ± 0.23^de^	14.87 ± 0.03^e^	57.33 ± 0.12^b^	22.24 ± 0.36^ab^	5.56 ± 0.56^fg^	335 ± 1.36^f^
4	9	1400	0.119 ± 0.06^ef^	−16.57 ± 0.33^f^	35.22 ± 0.11^a^	50.66 ± 0.31^c^	11.25 ± 0.33^i^	2.86 ± 0.13^h^	559 ± 1.33^a^
5	9	875	0.133 ± 0.02^def^	−14.36 ± 0.26^de^	16.76 ± 0.13^cd^	45.56 ± 0.66^e^	13.06 ± 0.22^gh^	24.22 ± 0.33^ab^	412 ± 1.24^cd^
6	9	875	0.135 ± 0.02^def^	−14.63 ± 0.52^e^	16.00 ± 0.16^d^	46.12 ± 0.57^de^	13.78 ± 0.27^g^	24.02 ± 0.66^ab^	408 ± 0.98^d^
7	6	875	0.248 ± 0.03^bc^	−14.27 ± 0.27^de^	9.22 ± 0.03^h^	45.61 ± 0.28^e^	33.2 ± 0.67^a^	11.97 ± 0.12^de^	213 ± 1.16^h^
8	9	875	0.129 ± 0.06^ef^	−14.60 ± 0.33^e^	17.75 ± 0.21^bc^	45.55 ± 0.67^e^	12.59 ± 0.21^gh^	23.89 ± 0.14^b^	398 ± 1.48^d^
9	9	350	0.152 ± 0.11^d^	−16.70 ± 0.09^f^	11.67 ± 0.13^fg^	66.92 ± 1.23^a^	18.86 ± 0.13^de^	2.55 ± 0.12^h^	366 ± 1.65^e^
10	7	500	0.311 ± 0.21^b^	−15.23 ± 0.04^e^	14.71 ± 0.15^e^	57.37 ± 1.01^b^	22.27 ± 0.36^ab^	5.65 ± 0.03^fg^	184 ± 1.58^h^
11	12	875	0.131 ± 0.03^def^	−25.10 ± 0.02^h^	17.74 ± 0.12^bc^	52.88 ± 1.12^c^	23.09 ± 0.56^ab^	6.28 ± 0.05^f^	470 ± 2.12^b^
12	9	875	0.138 ± 0.04^def^	−14.20 ± 0.01^de^	18.01 ± 0.08^bc^	46.22 ± 0.87^de^	14.11 ± 0.07^g^	22.56 ± 0.21^b^	405 ± 2.48^d^
13	9	875	0.131 ± 0.02^def^	−15.03 ± 0.13^e^	17.55 ± 0.06^bc^	45.44 ± 0.66^e^	13.98 ± 0.06^g^	25.12 ± 0.48^a^	421 ± 2.32^c^
14	7	1250	0.242 ± 0.08^c^	−12.33 ± 0.12^cd^	18.83 ± 0.03^bc^	47.07 ± 0.23^de^	20.59 ± 0.12^cd^	13.5 ± 0.54^c^	227 ± 1.72^g^

*Note*: Values are given as mean ± standard deviation from triplicate determinations. Means within the same column with different superscript letters are significantly different (*P* < 0.05). C1, hazelnut protein isolates at pH 4.5; C2, hazelnut protein isolates at pH 7.0 (neutral pH).

#### Zeta potential

Zeta potential arises from the electrostatic interactions between particles and the liquid in which they are suspended. Zeta potential indicates the charge stability and electrokinetic potential of protein aggregates. A correlation exists between zeta potential and nanoparticle stability. The control HPI exhibited nearly neutral zeta potential (+0.14 mV), indicating poor intrinsic stability. It was determined that pH is a more decisive factor in determining zeta potential than pressure. However, under certain conditions, high pressure combined with high pH significantly improved system stability (*P* < 0.05). For instance, at pH 11 and 1250 bar, the zeta potential measured −20.5 mV, while at the same pH but 500 bar, it dropped to −14.03 mV, suggesting that high pressure supports the achievement of more negative zeta potential values (Table 5). At pH 9, zeta potential values were −16.7 mV at 350 bar and −16.57 mV at 1400 bar, indicating no substantial change, which suggests that the pressure effect is not as pronounced as that of pH. As pH increased, zeta potential became more negative, especially under alkaline conditions, likely due to the reorganization of ionizable groups on protein surfaces and the alteration of electrostatic charge distribution (Fig. 3). The combination of high pH and pressure induces conformational changes in proteins, which generally increase the magnitude of negative zeta potential values and, in turn, influence inter‐particle interactions and colloidal stability. Several studies support this trend, reporting that pressure treatments enhance the absolute zeta potential of plant protein isolates. For instance, Ma *et al*.^24^ observed that chickpea protein isolates subjected to 30–150 MPa exhibited absolute zeta potential values consistently above 30 mV, whereas Zhao *et al*.^34^ reported that quinoa protein isolates increased from 15.77 to 43.73 mV with increasing pressure. Similarly, Bahmanyar *et al*.^35^ found that pea protein isolates exhibited a shift from near‐neutral values (1 mV) to markedly negative values (−13.4 mV) at 100 MPa. Collectively, these findings indicate that pressure promotes the disruption of aggregates and the unfolding of protein molecules, thereby exposing hydrophobic and charged residues. The increased surface charge density enhances electrostatic repulsion, thereby improving dispersion and stability.

#### Secondary structures

To investigate conformational changes in HPIs before and after high‐pressure processing, the secondary structure of protein isolates was determined using FTIR data (1600–1700 cm^−1^) (Table 5). The corresponding ANOVA is summarized in Table 6. The application of pressure resulted in significant changes in the secondary structures of the proteins (Table 6). The α‐helix content decreased with increasing pH and pressure (Fig. 3). For instance, sample C1 (pH 4.5, no pressure) showed an α‐helix content of 20.80%, while sample 11 (pH 12, 875 bar) showed a reduced value of 15.89%. This reduction suggests that alkaline conditions, combined with high‐pressure treatment, promote the unfolding of helical structures, likely due to electrostatic repulsion and the disruption of hydrogen bonds.^22^ The β‐sheet content remained relatively stable across most samples, ranging between 40.92% and 44.81%. However, a slight decrease was observed under extreme alkaline conditions (Fig. 3). The stability of β‐sheet structures indicates their resilience to moderate pH and pressure changes, which contributes to maintaining protein aggregation and gelation capacity.^36^ The persistent β‐sheet content may also support the formation of intermolecular interactions, which are essential for emulsion and foam stabilization. In contrast to α‐helix, both β‐turn and random coil contents generally increased under alkaline and high‐pressure conditions. For example, sample 2 (pH 11, 1250 bar) exhibited β‐turn and random coil values of 15.61% and 27.75%, respectively, compared to C1 (11.84% and 22.54%). The increase in these unstructured or flexible regions suggests partial protein unfolding and greater molecular flexibility, which can enhance solubility and surface activity.^36^ The rise in random coil content is particularly correlated with improved techno‐functional properties, such as emulsifying activity and foam capacity, as it facilitates better interaction at oil–water and air–water interfaces.^37^ These findings are consistent with recent studies on other plant proteins. Cheng *et al*.^38^ reported that pea protein isolate remained structurally stable at 60 MPa, showing no significant changes in β‐sheet, α‐helix, or random coil content. However, at higher pressures (120–240 MPa), β‐sheet and random coil structures increased at the expense of α‐helices, indicating partial unfolding and structural rearrangement. Similar trends were observed in walnut proteins, with high‐pressure processing treatment reducing β‐sheet and β‐turn contents and increasing random coil content.^39^ These transitions were attributed to cavitation during pressurization, which disrupts ordered structures and exposes sulfhydryl and hydrophobic groups. Collectively, these findings suggest that HPHT facilitates protein unfolding and disordering, thereby modulating both structural and functional properties.

**Table 6:** The effects of process parameters on the structural properties of hazelnut protein isolates

Factors	Average particle size (D50) (μm)		Zeta potential (mV)		α‐Helix (%)		β‐Sheet (%)		β‐Turn (%)		Random coil (%)		Surface hydrophobicity		
	Coefficient	*P*	Coefficient	*P*	Coefficient	*P*	Coefficient	*P*	Coefficient	*P*	Coefficient	*P*	Coefficient	*P*
	Modified quadratic		Quadratic		Linear		Linear		Linear		Linear		Quadratic		
Model	0.0744	0.0014	113.24	0.008	9.99	0.0077	5.5	0.013	4.69	0.0143	16.54	<0.0001	1.305E	0.0020	
A – pH	0.0557	0.0001	62.08	0.0022	1.52	0.1517	1.43	0.0903	4.44	0.9669	0.3911	0.1852	74 972	0.0005	
B – Pressure (bar)	0.0054	0.0443	1.43	0.5204	8.48	0.0039	4.06	0.0097	0.2508	0.0043	16.15	<0.0001	27 371	0.0095	
AB			21.95	0.0302									2970	0.2961	
A^2^	0.0132	0.0068	27.31	0.0189									24 859	0.0120	
B^2^			1.17	0.5604									45.39	0.8935	
Lack‐of‐fit	0.0095	0.0001	24.38	0.0006	4.43	0.3587	1.25	0.3127	5.32	0.0586	1.49	0.2543	18 620	0.0001	
Pure error	0.0001		0.995		2.6		3.33		0.7486		0.6636		395.33		
*R* ^2^	0.8793		0.8169		0.5871		0.5457		0.3678		0.8848		0.8728		
Adjusted‐*R* ^2^	0.843		0.7025		0.5121		0.4631		0.2529		0.8639		0.7934		

#### Hydrophobicity

Surface hydrophobicity is a key structural attribute influencing protein functionality, including solubility, emulsification, and foaming. In the present study, the control sample exhibited a surface hydrophobicity value of 120 units, whereas significant increases were observed following high‐pressure treatment. The unfolding process exposes both hydrophilic and hydrophobic groups. This enhancement can be attributed to the migration of hydrophobic amino acid residues from the protein interior to the surface. Consistent with these findings, previous studies have emphasized that surface hydrophobicity is highly sensitive to both pH and pressure, reflecting pressure‐induced structural rearrangements. For example, Yildiz and Yıldız^11^ demonstrated that quinoa proteins displayed a marked increase in hydrophobicity under alkaline conditions (from 98 to 174 at pH 12), which was further elevated to 198 after high‐pressure processing. This increase was attributed to the transition of proteins from compact structures to more open conformations in the presence of strong electrolytes, thereby exposing previously buried hydrophobic groups. Nevertheless, not all proteins exhibit this trend. Zhao *et al*.^40^ reported that jujube seed, hemp seed, and plum seed protein isolates showed reduced hydrophobicity at 90 MPa, a change attributed to pressure‐induced aggregation masking hydrophobic residues. Conversely, wolfberry protein isolates exhibited enhanced hydrophobicity following high‐pressure homogenization, associated with protein unfolding and the exposure of hydrophobic domains. Collectively, these findings suggest that the response of surface hydrophobicity to high pressure is protein‐specific, and the balance between unfolding, which exposes hydrophobic residues, and aggregation, which conceals them, ultimately determines the observed effect.

In this study, the highest value (559 units) was recorded at pH 9 under 1400 bar, while a similarly high value (420 units) was observed at pH 9 and 875 bar. This indicates that a moderate alkaline pH, combined with high pressure, effectively promotes structural rearrangements, thereby exposing hydrophobic regions. The effects of both pH and pressure on surface hydrophobicity were statistically significant (*P* < 0.05) (Table 6). Elevated pH and pressure result in partial denaturation, exposing hydrophobic groups and enhancing protein–lipid interactions, which are crucial for technological properties such as emulsification and foam stability. As the pH approaches alkaline values, hydrophobic groups migrate to the surface, while increased pressure leads to the unfolding and exposure of buried residues (Fig. 3). Previous studies have also reported significant increases in the surface hydrophobicity of plant protein isolates due to changes in pH. For instance, Sun *et al*.^40^ observed an increase in surface hydrophobicity  with the increasing alkalinity, although methodological differences in probe sensitivity may partly explain the higher absolute values compared with the present study.

### Optimization, validation and characterization of the optimum sample

Optimization was carried out considering solubility, EAI, ESI, and apparent viscosity (*η*_50_) as response variables. The optimum conditions for encapsulation were identified as pH 12 and 875 bar, yielding the highest combined values of solubility, EAI, ESI, and *η*_50_ (overall desirability = 0.688). The deviations between theoretical and experimental results are given in Table 7. The deviations are lower than 5% which means experimental results correct the predictivity of the models.

**Table 7:** Comparison of experimental and predicted values obtained under optimum conditions for hazelnut protein isolates (desirability = 0.688)

Sample	A: pH	B: Pressure (bar)	C: Protein ratio (%)	Solubility (%)	Emulsion activity (m^2^ g^−1^)	Emulsion stability (%)	Apparent viscosity (Pa s)
Predicted values	12	875	35	84.44 ± 0.04^a^	33.12 ± 0.03^a^	31.78 ± 0.09^a^	6.44 ± 0.05^a^
Experimental values	12	875	35	81.35 ± 0.14^a^	31.08 ± 0.13^a^	30.38 ± 0.02^a^	6.12 ± 0.07^b^
Deviation (%)				3.6%	3.95%	4.40%	4.96%

*Note*: Values are given as mean ± standard deviation from triplicate determinations. Means within the same column with different superscript letters are significantly different (*P* < 0.05).

#### Allergenicity

The immunoreactive protein content of the modified HPI was assessed using a commercial Hazelnut Allergen ELISA kit. The allergenic potential of food proteins is a critical concern in food safety and consumer health. The allergenicity of HPI produced under optimal conditions was reduced by 49% compared with the control. Similar trends have been reported for other plant protein isolates, where HPHT decreases allergenicity primarily by disrupting conformational epitopes. For instance, Li *et al*.^41^ demonstrated that soy protein isolate treated at 300 MPa for 15 min exhibited a 48.6% reduction in allergenicity relative to its native form. Consistently, Yao *et al*.^42^ observed a pressure‐dependent decline in the allergenicity of wheat gluten, with reductions ranging from 200 to 400 MPa, and a maximum decrease of 72.2% at 400 MPa. The observed reduction in immunoglobulin G (IgG) reactivity, however, is a promising indicator that the treatment may have modified potential allergenic epitopes, a hypothesis that requires confirmation through specific IgE‐binding assays or other advanced immunochemical methods.

#### Microstructure

**Figure 4:**
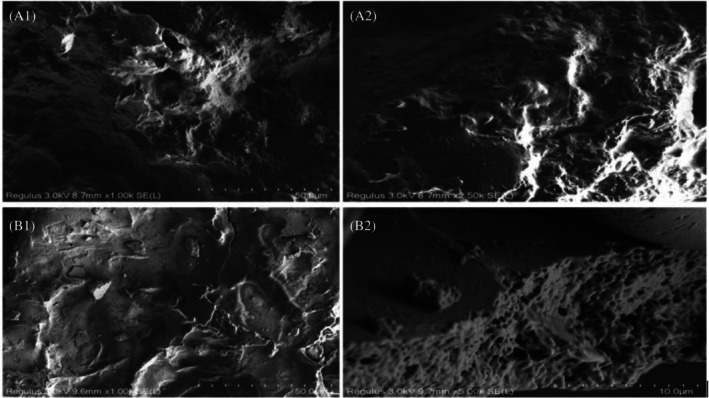
Scanning electron microscopy (SEM) image of unmodified (control) hazelnut protein isolate (HPI) sample (A1: 500× magnification; A2: 2000× magnification), SEM image of optimum modified HPI (pH 12, 872 bar) sample (B1: 500× magnification; B2: 2000× magnification).

SEM images of the control HPI and optimum HPI (pH 12, 875 bar) samples are shown in Fig. 4. The control sample exhibited a relatively compact morphology with aggregated structures and limited porosity. In contrast, the optimum HPI exhibited a rougher and more heterogeneous surface with numerous cracks and pores, indicating disruption of aggregates under HPHT conditions. These morphological transitions suggest that cavitation and shear forces caused fragmentation of protein networks and promoted the formation of smaller, porous, and irregular structures. Consistent with our observations, previous studies have reported similar alterations in plant proteins subjected to high pressure. For instance, Bahmanyar *et al*.^35^ found that untreated pea protein isolate exhibited spherical aggregates with large pores (~50 μm), which were transformed into smaller heterogeneous structures (~5 μm) after high pressure treatment (60–100 MPa). Similarly, Yang *et al*.^39^ observed that walnut proteins treated with high pressure exhibited rougher microstructures, with enlarged pores and cracks, compared to the control. These structural modifications are generally attributed to aggregate breakdown and partial denaturation, leading to more porous and compact morphologies under pressure.

### Using optimum HPI as a wall material: probiotic encapsulation

When the effects of inlet temperature and wall material composition on probiotic encapsulation were examined, it was found that survival decreased significantly as inlet temperature increased (*P* < 0.05). The highest survival was observed at 130 °C with a 1:1 HPI/MD ratio (7.96 log CFU mL^−1^), whereas the lowest survival was observed at 160 °C with HPI alone (5.7 log CFU mL^−1^). This indicates that using HPI as the sole carrier at elevated temperatures reduces probiotic viability (Table 8). Previous studies have demonstrated that the type and combination of carrier materials directly affect the survival of probiotic microorganisms during spray drying. Hu *et al*.^42^ encapsulated *Lactobacillus plantarum* using soy protein isolate and pectin, reporting that viability decreased from 9.69 to 9.11 log CFU g^−1^ with soy protein isolate alone, and from 9.65 to 9.35 log CFU g^−1^ with the soy protein isolate–pectin (1:1) combination. The improved protection was attributed to pectin filling cracks and voids in soy protein isolate‐based microcapsules, making the structure more compact and creating a more effective barrier. Yu *et al*.^43^ encapsulated *Lactobacillus paracasei* with succinylated chickpea protein, showing that viability increased from 40.84% with untreated protein to 48.74% after protein modification. These findings emphasize that both carrier composition and structural modifications significantly impact probiotic stability.

**Table 8:** Survival rates of *Lactobacillus acidophilus* after spray drying and *in vitro* digestion

Inlet air temperature (°C)	Feed rate (mL dk^−1^)	Wall material proportion (HPI/MD)	Outlet temperature (°C)	Initial (log CFU mL^−1^)	After spray drying (log CFU g^−1^)	*In vitro* gastric digestion (log CFU mL^−1^)	*In vitro* ıntestinal digestion (log CFU mL^−1^)	Gastric survival (%)	Intestinal survival (%)
—	—	Free cell	—	10.23 ± 0.02	ND	2.3 ± 0.04^a^	2.05 ± 0.03^a^	ND	ND
130	10	1:1	54	9.76 ± 0.01	7.96 ± 0.02^a^	7.22 ± 0.04^a^	6.52 ± 0.08^a^	74.05 ± 0.06^a^	66.80 ± 0.03^a^
130	10	0:1	61	9.77 ± 0.03	7.47 ± 0.02^c^	4.95 ± 0.06^e^	4.28 ± 0.02^e^	50.66 ± 0.03^e^	43.80 ± 0.06^e^
130	10	1:0	57	9.76 ± 0.04	7.61 ± 0.03^b^	6.11 ± 0.03^c^	5.48 ± 0.06^c^	62.60 ± 0.12^c^	56.14 ± 0.05^c^
160	10	1:1	68	9.83 ± 0.05	7.12 ± 0.01^e^	6.89 ± 0.02^b^	6.10 ± 0.09^b^	70.09 ± 0.09^b^	62.05 ± 0.03^b^
160	10	0:1	68	9.83 ± 0.02	7.26 ± 0.02^d^	5.64 ± 0.06^d^	2.30 ± 0.12^f^	57.37 ± 0.02^d^	23.39 ± 0.02^f^
160	10	1:0	68	9.85 ± 0.03	5.7 ± 0.04^f^	5.60 ± 0.06^d^	4.80 ± 0.05^d^	56.85 ± 0.06^d^	48.73 ± 0.05^d^

*Note*: Values are given as mean ± standard deviation from triplicate determinations. Means within the same column with different superscript letters are significantly different (*P* < 0.05). ND, not determined. Gastric and intestinal survival rates were calculated according to the microorganism count after spray drying. HPI, hazelnut protein isolate; MD, maltodextrin.

The harsh gastric environment, characterized by high hydrogen ion concentration, pepsin activity, and osmotic stress, is known to damage cell integrity and cause substantial reductions in probiotic viability.^44^ In this study, *in vitro* gastric digestion assays confirmed a marked decline in viable counts across all treatments. The highest resistance was observed with the combined use of HPI and MD at 130 °C, resulting in a 7.22 log log CFU mL^−1^ count. This probiotic passage through the stomach indicates successful encapsulation, as such a quantity is sufficient to exert a probiotic effect in the intestine. In contrast, MD alone provided only moderate protection during spray drying at 160 °C and was insufficient to maintain viability under gastrointestinal conditions, resulting in substantial losses. HPI alone provided stronger protection than MD, although its efficacy slightly decreased at higher inlet temperatures, likely due to pressure‐ and heat‐induced structural alterations. Overall, these findings indicate that while MD is a poor carrier for maintaining probiotic viability under digestive stress, HPI exhibits superior protective functionality. Moreover, the combination of HPI and MD in a 1:1 ratio provided synergistic benefits, reducing viability loss during gastric digestion and highlighting its potential as an effective encapsulation matrix (Table 8).

## CONCLUSION

This study demonstrates that HPI derived from cold‐press extraction cake can be effectively valorized through HPHT (350–1400 bar) combined with pH shifting (pH 6.0–12.0). The modified HPI showed markedly improved solubility (up to 86.5%), emulsifying activity (33.12 m^2^ g^−1^), and foaming capacity (49.44%), which were linked to conformational reorganization (reduced α‐helix, increased random coil), enhanced surface hydrophobicity (from 120 to 559 units), and reduced particle size (from 391 μm to 0.13 μm). These structural modifications also decreased allergenic potential by 49%, suggesting disruption of conformational epitopes. Furthermore, the optimized HPI (pH 12, 875 bar) used as a wall material in combination with MD (1:1 ratio) achieved the highest *L. acidophilus* viability 7.96 log CFU mL^−1^ after spray drying and 7.22 log CFU mL^−1^ under simulated gastrointestinal conditions. Overall, the valorization of hazelnut meal into a multifunctional, hypoallergenic protein ingredient highlights both functional and sustainability benefits, offering promising applications in the formulation of functional foods and probiotic delivery systems. While this study demonstrates that combining high pH with HPHT effectively enhances the functional properties of HPI, it is crucial to address the potential safety implications of alkaline processing. As rightly noted, prolonged exposure to high hydroxide ion concentrations (particularly at elevated temperatures) can lead to chemical modifications in amino acids, most notably the formation of lysinoalanine (LAL). Future work should explicitly quantify the levels of LAL and other potential alkali‐induced compounds (e.g., lanthionine) in the modified HPI to ensure its full safety for food applications. This would be a critical step before any potential commercial adoption of this technology.

## AUTHOR CONTRİBUTİONS

Ilyas Atalar: conceptualization, methodology, supervision, writing – original draft. Hatice Elen: writing – original draft, formal analysis. Osman Gül: writing – review and editing. formal analysis. Abdullah Kurt: writing – review and editing, formal analysis. Muhammet Irfan Aksu: writing – review and editing. Nevzat Konar: writing – review and editing. conceptualization.

## CONFLICT OF INTEREST

The authors declare no conflict of interest and no competing financial interest.

## Data Availability

The data will be available from the corresponding author.
